# Trimethylamine N-Oxide and White Matter Hyperintensity Volume Among Patients With Acute Ischemic Stroke

**DOI:** 10.1001/jamanetworkopen.2023.30446

**Published:** 2023-08-23

**Authors:** Naruchorn Kijpaisalratana, Zsuzsanna Ament, Matthew B. Bevers, Varun M. Bhave, Ana-Lucia Garcia Guarniz, Catharine A. Couch, M. Ryan Irvin, W. Taylor Kimberly

**Affiliations:** 1 Center for Genomic Medicine, Massachusetts General Hospital, Harvard Medical School, Boston; 2 Department of Neurology, Massachusetts General Hospital, Boston; 3 Division of Neurology, Department of Medicine, Faculty of Medicine, Chulalongkorn University, Bangkok, Thailand; 4 Division of Academic Affairs, Faculty of Medicine, Chulalongkorn University, Bangkok, Thailand; 5 Divisions of Stroke, Cerebrovascular and Critical Care Neurology, Brigham and Women’s Hospital, Boston, Massachusetts; 6 Harvard Medical School, Boston, Massachusetts; 7 Department of Epidemiology, School of Public Health, University of Alabama at Birmingham

## Abstract

**Importance:**

Although increasing evidence suggests that trimethylamine N-oxide (TMAO) is associated with atherosclerosis, little is known about whether TMAO and its related metabolites (ie, choline, betaine, and carnitine) are associated with small vessel disease.

**Objective:**

To evaluate the association between TMAO and its related metabolites with features of cerebral small vessel disease, including white matter hyperintensity volume (WMHV) and acute lacunar infarction.

**Design, Setting, and Participants:**

This cross-sectional study included patients enrolled in the Specialized Programs of Translational Research in Acute Stroke biorepository. The registry included 522 patients with acute ischemic stroke who were 18 years or older who presented at the Massachusetts General Hospital or Brigham and Women’s Hospital within 9 hours after onset between January 2007 and April 2010. The analyses in this study were conducted between November 2022 and April 2023.

**Exposures:**

Plasma TMAO, choline, betaine, and carnitine were measured by liquid chromatography–tandem mass spectrometry.

**Main Outcomes and Measures:**

WMHV was quantified by a semiautomated approach using signal intensity threshold with subsequent manual editing. Ischemic stroke subtype was classified using the Causative Classification System.

**Results:**

Among 351 patients included in this study, the mean (SD) age was 69 (15) years; 209 patients (59.5%) were male and had a median (IQR) admission National Institute of Health Stroke Scale of 6 (3-13). The magnetic resonance imaging subgroup consisted of 291 patients with a mean (SD) age of 67 (15) years. Among these, the median (IQR) WMHV was 3.2 (1.31-8.4) cm^3^. TMAO was associated with WMHV after adjustment for age and sex (β, 0.15; 95% CI, 0.01-0.29; *P* < .001). TMAO remained significant in a multivariate analysis adjusted for age, sex, hypertension, diabetes, and smoking (β, 0.14; 95% CI, 0-0.29; *P* = .05). TMAO was associated with lacunar stroke but not other ischemic stroke subtypes in a model adjusted for age, sex, hypertension, diabetes, and smoking (OR, 1.67; 95% CI, 1.05-2.66; *P* = .03).

**Conclusions and Relevance:**

In this observational study, TMAO was associated with cerebral small vessel disease determined by WMHV and acute lacunar infarction. The association was independent of traditional vascular risk factors.

## Key Points

**Question:**

Are trimethylamine N-oxide (TMAO) and its related metabolites (ie, choline, betaine, and carnitine) associated with white matter hyperintensity volume (WMHV) and acute lacunar infarction?

**Findings:**

In a cohort of patients with acute ischemic stroke, TMAO was associated with WMHV and acute lacunar infarction but not other stroke subtypes. The association was independent of other traditional vascular risk factors.

**Meaning:**

These findings suggest that TMAO is associated with imaging and clinical features of cerebral small vessel disease, including white matter hyperintensities and acute lacunar infarction.

## Teaser

This cross-sectional study examines the association between trimethylamine-N-oxide (TMAO) and its related metabolites with small vessel disease and white matter hyperintensity volume among adults with acute ischemic stroke.

## Introduction

Cerebral small vessel disease (CVSD) is a leading cause of vascular cognitive impairment^1^ and accounts for a quarter of ischemic stroke cases.^2^ In addition, it also contributes to gait impairment^3^ and mood disturbances.^4^ CVSD is associated with a variety of neuroimaging findings, including lacunes, recent small subcortical infarcts, white matter hyperintensity volume (WMHV), enlarged perivascular spaces, cerebral microbleeds, and cortical microinfarcts.^2,5^ CVSD is a substantial modifier of brain health and is responsible for significant cognitive and functional disability worldwide,^5^ due to an increased risk of stroke, dementia, and mortality.^6^ Despite the widespread impact of the disease, few effective treatments to delay disease progression are available.^7^ This is at least partly due to a limited understanding of the pathogenesis of the disease.

Trimethylamine N-oxide (TMAO) has recently garnered attention due to its potential role in cardiovascular and cerebrovascular diseases.^8,9^ TMAO is a dietary gut microbiome-related metabolite^8^ generated by the oxidation of trimethylamine (TMA) by flavin-containing monooxygenases (FMOs).^10^ In humans, TMA is obtained primarily from the bacterial metabolism of dietary choline, betaine, and L-carnitine, which is highly abundant in red meat.^11^ An increasing body of evidence suggests that TMAO is associated with major adverse cardiovascular events.^12,13^

White matter hyperintensity (WMH) is a common sign and the most well-characterized imaging feature of CVSD.^5,14^ In this study, we sought to determine the role of TMAO and its related precursor metabolites, including choline, betaine, and carnitine, in cerebral small vessel disease. The objective of this study is to assess the association between these plasma metabolites and the WMHV. Because acute lacunar infarction is a common clinical manifestation of CVSD, we also examined the association between metabolites that demonstrated an association with WMHV and acute lacunar infarction.

## Methods

This cross-sectional study was approved by the Mass General Brigham (formerly Partners Healthcare) institutional review board. Written informed consent was obtained from all patients or their legal representatives prior to enrollment. This study followed the Strengthening the Reporting of Observational Studies in Epidemiology (STROBE) reporting guideline.

### Study Design and Population

The study design of the Specialized Programs of Translational Research in Acute Stroke (SPOTRIAS) Network has been described in detail elsewhere.^15,16,17^ In brief, 522 patients with acute ischemic stroke who were 18 years or older and presented at the Massachusetts General Hospital or Brigham and Women’s Hospital within 9 hours of stroke onset between January 2007 and April 2010 were consecutively enrolled. Patients were eligible if the National Institute of Health Stroke Scale (NIHSS) was 1 or higher.

In this study, 2 patients were excluded because there were absent details of stroke subtype, 9 patients had a discharge diagnosis of transient ischemic attack, 14 patients had a discharge diagnosis of nonstroke, and 136 patients did not have plasma available for metabolites measurement. Ethylenediaminetetraacetic acid (EDTA) blood samples from the patients were obtained at admission (corresponding to mean [SD] 7.1 [3.3] hours after last seen well time). Plasma was separated after centrifugation and stored at −80 °C until analysis.

### Stroke Subtype Classification

The ischemic stroke subtype in the SPOTRIAS was classified based on the Causative Classification System (CCS). This is the web-based system and the automated version of the evidence-based Stop Stroke Study TOAST (SSS-TOAST) causative classification algorithm. The detail of the stroke classification method has been described in detail previously.^18,19^ The algorithm requires clinical, diagnostic, and epidemiologic data to classify ischemic stroke into 5 subtypes, including large artery atherosclerosis, cardio-aortic embolism, small artery occlusion, other causes, and undetermined causes.

### WMH Analysis

Magnetic resonance imaging (MRI) was obtained as part of routine clinical care in a subset of the study population within 96 hours from symptom onset on 1.5T MRI scanners. The volumetric algorithm for quantifying the WMHV has been previously reported^17^ and used multiple MRI sequences, including axial T2 fluid-attenuated inversion recovery, sagittal T1, and diffusion-weighted imaging. The WMH map was derived from an overlap between a semiautomated signal intensity threshold and supratentorial region-of-interest outlines with subsequent manual editing. The WMHV was derived by doubling the WMHV from the hemisphere contralateral to acute ischemic stroke to avoid the integration of the hyperintensity signal resulting from acute brain infarction. Structures prone to T2 hyperintensity artifact, including basal ganglia, thalamus, mesial temporal areas, corticomedullary junction line, and ventricular lining, were excluded from this analysis.^17,20^ The WMHV was normalized to the intracranial area and natural log-transformed before analysis, similar to a previous SPOTRIAS study.^17^

### TMAO and Related Metabolites Measurement

Plasma TMAO and its related metabolites, including choline, betaine, and carnitine, were measured by liquid chromatography with tandem mass spectrometry using previously described methods.^21,22^ In brief, polar metabolites were extracted by precipitating protein from 30 μL of plasma EDTA. Metabolites were separated by dual infinity II 1290 high-performance liquid chromatography pumps on an Xbridge Amide column (2.1 × 100 mm 3.5 μm) and were detected by a 6495 triple-quadrupole mass spectrometer. Peaks were integrated and analyzed using MassHunter QQQ Quantitative Analysis software. The sample order was randomized between patients and the serial samples within patients were measured in adjacent injections in random order to minimize the batch effects. In addition, the metabolite measurements were quality controlled and normalized across batches by including human pooled plasma samples at regular intervals of every 10 injections. All metabolite level values were rank-based inverse normal transformed before statistical analysis since they did not conform to a normal distribution.^22,23,24^

### Statistical Analyses

Analyses for this study were performed between November 2022 and April 2023. Baseline characteristics were presented as mean (SD) for normally distributed continuous variables or median (IQR) for continuous variables with deviation from normality. Categorical variables were presented as frequency and percentage. The baseline characteristics between the overall cohort and the MRI subgroup were compared using *t* test, Wilcoxon rank-sum test, and χ^2^ for normally distributed continuous, nonnormal continuous, and categorical variables, respectively. Linear regression was performed to determine the association between TMAO and its related metabolites and WMHV in a univariate analysis and a model adjusted for age and sex. In addition, a model adjusted for vascular risk factors, such as hypertension, diabetes, and current smoking status, was also evaluated. A Bonferroni adjusted *P* value was used to account for the multiple testing of 4 metabolites. A linear mixed model with repeated measures using TMAO as the outcome was used as a sensitivity analysis and included the subset of patients with plasma samples collected at a second time point approximately 48 hours after stroke onset. Logistic regression modeling was performed to assess the association between the metabolite and the stroke subtypes. All tests were 2-sided with a significant threshold of *P* < .05. All statistical analyses were performed using Stata version 17.0 (StataCorp). The Figure was generated by GraphPad Prism version 9.5.1 (GraphPad Software).

**Figure.**
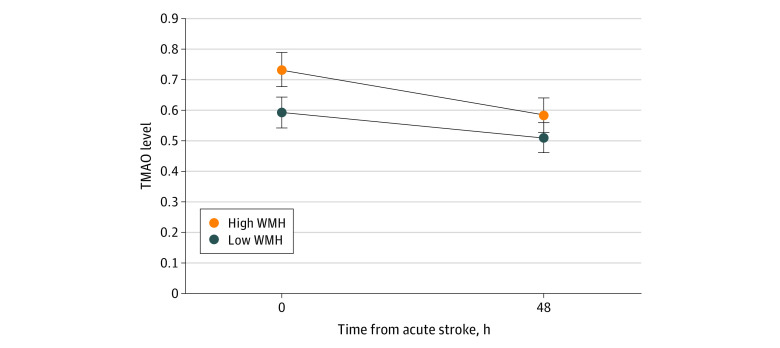
Plasma Trimethylamine-N-Oxide (TMAO) Level Over Time in Patients With High and Low White Matter Hyperintensity (WMH)

## Results

### Study Population and Baseline Characteristics

Among 351 patients included in this study, the mean (SD) age was 69 (15) years; 209 patients (59.5%) were male and had a median (IQR) admission National Institute of Health Stroke Scale (NIHSS) of 6 (3-13). The MRI subgroup consisted of 291 patients with a mean (SD) age of 67 (15) years. Among these, the median (IQR) WMHV was 3.2 (1.31-8.4) cm^3^. Hypertension occurred in 255 (72.6%). Cardioembolic stroke (175 [49.9%]) was the most common stroke subtype in the study cohort, followed by large vessel atherosclerosis (68 [19.4%]), undetermined (63 [17.9%]), small vessel disease (27 [7.7%]), and other determined etiology (18 [5.1%]). Patients who did not have MRI were older and had more severe strokes (Table 1). There were no significant differences in baseline characteristics between the study population and the MRI subgroup (eTable in Supplement 1).

**Table 1.** Baseline Characteristics^a^

Characteristics	Patient, No. (%)		
	MRI subgroup (291)	No MRI subgroup (60)	*P* value
Age, mean (SD), y	67 (15)	76 (12)	<.001
Sex			
Male	178 (61.17)	31 (51.67)	.17
Female	113 (38.83)	29 (48.33)	
Stroke risk factors			
Hypertension	212 (72.85)	43 (71.67)	.85
Diabetes	58 (19.93)	17 (28.33)	.15
Coronary artery disease	76 (26.12)	23 (38.33)	.06
Currently smoking	58 (19.93)	6 (10.17)	.08
Hyperlipidemia	140 (48.11)	29 (48.33)	.98
Prior stroke	57 (19.59)	13 (21.67)	.71
Stroke subtypes			
Large vessel atherosclerosis	63 (21.65)	5 (8.33)	.03
Small vessel disease	26 (8.93)	1 (1.67)	
Cardioembolic	137 (47.08)	38 (63.33)	
Other	15 (5.15)	3 (5.00)	
Undetermined	50 (17.18)	13 (21.67)	
Admission NIHSS, median (IQR)	5 (2-12)	12 (6-18)	<.001
90-d mRS, median (IQR)	1 (1-3)	4 (2-6)	<.001
WMH, median (IQR)	−3.2 (1.3 to 8.4)	NA	NA

Abbreviations: MRI, magnetic resonance imaging; mRS, modified Rankin Scale; NA, not available; NIHSS, National Institute of Health Stroke Scale; WMH, white matter hyperintensity.

^a^ Number of missing observations for each variable (n): age (6), smoking (1), admission NIHSS (1), and mRS (40).

### TMAO and Its Related Metabolites in Association With WMH

First, we examined the association between plasma TMAO and related metabolites (choline, betaine, and carnitine) and WMHV. TMAO was associated with WMHV in univariate analysis (β, 0.34; 95% CI, 0.18-0.49; *P* <.001) and a model adjusted for age and sex (β, 0.15; 95% CI, 0.01-0.29; *P* = .04) (Table 2). Of the TMAO-related metabolites, choline was associated with WMHV in univariate but not in the age- and sex-adjusted analysis. Betaine and carnitine were not associated with WMHV (Table 2).

**Table 2.** TMAO and Its Related Metabolites Association With White Matter Hyperintensity

Characteristic	Model 1^a^		Model 2^b^	
	β (95% CI)	*P* value	β (95% CI)	*P* value
TMAO	0.34 (0.18 to 0.49)	<.001^c^	0.15 (0.01 to 0.29)	.04
Choline	0.17 (0.02 to 0.33)	.03	0.03 (−0.11 to 0.17)	.71
Betaine	0.08 (−0.07 to 0.24)	.30	0.06 (−0.08 to 0.20)	.38
Carnitine	−0.02 (−0.18 to 0.15)	.85	0.02 (−0.13 to 0.16)	.82

Abbreviation: TMAO, trimethylamine N-oxide.

^a^ Model 1: metabolite (univariate).

^b^ Model 2: age + sex + metabolite.

^c^ *P* value less than the Bonferroni-corrected critical value.

We also performed a sensitivity analysis that included the subset of patients with plasma samples available at a second time point approximately 48 hours after stroke onset (220 patients [62.7%]). In a repeated measures mixed-effects model, the association between TMAO and WMHV remained significant in both a univariate (β, 0.16; 95% CI, 0.09-0.23; *P* <.001) and a model adjusted for age and sex (β, 0.09; 95% CI, 0.01-0.18; *P* = .03). The Figure demonstrated the levels of TMAO over time between patients with high WMHV and low WMHV using the median volume of WMH as the cutoff point. The mean (SD) relative levels of TMAO were higher in patients with high WMHV (0.10 [0.94]) compared with low WMHV (−0.32 [0.94]) at baseline (*P* <.001) and had a trend of higher levels in high WMHV group at 48 h (high WMHV, 0.06 [0.94] vs low WMHV, −0.21 [0.91]; *P* = .05). However, there were no significant changes regarding the TMAO level over time in high WMHV (baseline, 0.13 [0.94] vs 48 h, 0.11 [1.02]; *P* = .80), low WMHV (baseline, −0.23 [0.97] vs 48 h, −0.81 [1.02]; *P* = .13), and overall patients (baseline, −0.09 [0.97] vs 48 h, −0.02 [0.99]; *P* = .28).

### Factors Associated With WMHV

Next, we explored the association between TMAO and WMHV along with other traditional risk factors known to be associated with WMH, including age, hypertension, diabetes, and smoking.^25^ In addition to TMAO, age (β, 0.05; 95% CI, 0.04-0.05; *P* < .001) and hypertension (β, 0.95; 95% CI, 0.63-1.27; *P* <.001), were associated with WMHV in a univariate analysis. Age (β, 0.04; 95% CI, 0.03-0.05; *P* <.001), hypertension (β, 0.42; 95% CI, 0.09-0.74; *P* = .01), and TMAO (β, 0.14; 95% CI, 0.0003-0.287; *P* = .05) remained significant in a multivariate analysis adjusted for age, sex, hypertension, diabetes, and smoking (Table 3).

**Table 3.** Univariate and Multivariate Estimators of White Matter Hyperintensity

Estimators	Univariate		Multivariate^a^	
	β (95% CI)	*P* value	β (95% CI)	*P* value
Age	0.05 (0.04 to 0.05)	<.001	0.04 (0.03 to 0.05)	<.001
Sex				
Male	−0.31 (−0.61 to 0)	.05	−0.16 (−0.43 to 0.11)	.25
Female	0.31 (0 to 0.61)	.05	0.16 (−0.11 to 0.43)	.25
Trimethylamine N-oxide	0.34 (0.18 to 0.49)	<.001	0.14 (0 to 0.287)	.05
Hypertension	0.95 (0.63 to 1.27)	<.001	0.42 (0.09 to 0.74)	.01
Diabetes	0.14 (−0.24 to 0.52)	.46	−0.08 (−0.42 to 0.26)	.63
Currently smoking	−0.19 (−0.57 to 0.19)	.32	0.11 (−0.22 to 0.44)	.52

^a^ Multivariate analysis model: age + sex + hypertension + diabetes + currently smoking.

### TMAO Associated With Lacunar Infarction

We reasoned that TMAO may also be associated with certain ischemic stroke subtypes. We evaluated the association between TMAO and the ischemic stroke subtypes classified by CCS. TMAO was associated with acute lacunar infarction, but not other ischemic stroke subtypes in both the age- and sex-adjusted model (OR, 1.60; 95% CI, 1.02-2.50; *P* = .04) and in the model adjusted for age, sex, hypertension, diabetes, and smoking (OR, 1.67; 95% CI, 1.05-2.66; *P* = .03) (Table 4).

**Table 4.** Association Between Trimethylamine N-Oxide Association and Stroke Subtypes

Trimethylamine N-oxide	Base model^a^		Adjusted model^b^	
	OR (95% CI)	*P* value	OR (95% CI)	*P* value
Lacunar stroke	1.60 (1.02-2.50)	.04	1.67 (1.05-2.66)	.03
Large vessel atherosclerosis	0.87 (0.65-1.17)	.36	0.88 (0.65-1.18)	.39
Cardioembolic stroke	1.02 (0.80-1.29)	.90	0.99 (0.78-1.27)	.95
Others	0.86 (0.49-1.51)	.60	1.02 (0.55-1.87)	.96
Undetermined	0.92 (0.68-1.25)	.60	0.90 (0.66-1.22)	.49

Abbreviation: OR, odds ratio.

^a^ Base model: age + sex + metabolite.

^b^ Adjusted model: age + sex + hypertension + diabetes + currently smoking + metabolite.

## Discussion

In this cross-sectional study, we found that TMAO levels were associated with the volume of WMH among patients with acute ischemic stroke. The association between TMAO and WMHV remained after adjusting for vascular risk factors, including hypertension, diabetes, and smoking. In addition, TMAO levels were associated with acute lacunar infarction but not other stroke subtypes. Taken together, our study demonstrated an association between TMAO and common features of CVSD, including WMH and lacunar infarction.

Many observational and experimental studies have demonstrated a causal relationship between TMAO or its related metabolite and an increased risk of cardiovascular diseases.^11,12,13,26,27^ Recent meta-analyses also demonstrated that higher plasma TMAO levels are associated with an increased risk of stroke.^28,29^ The association between TMAO and stroke was present for first-time stroke^30^ and recurrent stroke.^31^ Furthermore, the stroke severity as determined by NIHSS or infarct volume was associated with TMAO level.^32,33^ However, there are relatively few studies focusing on TMAO and CVSD. Elevated TMAO levels were associated with an increased risk of recurrent lacunar infarction but not in other ischemic stroke subtypes.^31^ Although there are observational studies that demonstrated the association between cerebral small vessel imaging markers determined by WMH and TMAO levels,^34,35^ none of these evaluated the association between TMAO and the clinical presentation of CVSD.

The underlying mechanisms relating TMAO to both cardiovascular and cerebrovascular disease are poorly understood. Preclinical studies have evaluated several potential mechanisms, which are mainly related to atherosclerosis.^8,9^ TMAO promotes the upregulation of multiple macrophage scavenger receptors leading to foam cell formation, which is linked to the development of atherosclerosis.^13^ TMAO also plays an important role in regulating cholesterol and sterol metabolism. Dietary supplement with TMAO or choline in mice reduces reverse cholesterol transport, which is the mechanism for removing excess cholesterol from peripheral tissue back to the liver.^11^ Furthermore, TMAO increases the risk of thrombotic events through platelet hyperactivity.^36^

Another potential mechanism by which TMAO is linked to vascular diseases is endothelial dysfunction. Several studies have demonstrated that TMAO promotes inflammation and endothelial dysfunction through different signaling pathways. TMAO has been shown to cause endothelial dysfunction by activating the NLRP3 inflammasome signaling pathway in both in vitro and in vivo studies.^37,38^ TMAO activates inflammatory cytokines and adhesion molecules, in part, through the mitogen-activated protein kinase and nuclear factor-κB signaling pathways.^39^ A study^40^ including healthy humans suggested that TMAO promotes age-related endothelial dysfunction via oxidative stress.

Endothelial dysfunction plays a major role in the pathogenesis of CVSD.^5,7,25,41^ In particular, WMH results from diffuse endothelial dysfunction with increased blood-brain barrier permeability. This causes the leakage of plasma materials, including fluids and proteins, into the vessel wall and perivascular space, leading to inflammation, arteriolar wall thickening, and stiffening.^5,25,42^

The link between atherosclerosis in different vascular territories and CVSDs has also been demonstrated in several observational studies. The presence of coronary artery plaque is associated with larger WMHV in healthy individuals.^14^ Periventricular WMH in elderly individuals is associated with aortic atherosclerosis during midlife.^43^ Carotid atherosclerosis determined by higher common carotid intima to media wall thickness and carotid plaques is associated with WMH in the Rotterdam study.^44^ Intracranial atherosclerosis is associated with WMHV among healthy participants without a history of stroke.^45^

Although TMAO has been shown to be associated with several vascular risk factors, such as hypertension^46,47^ and diabetes,^48,49^ the association between TMAO and both WMH and lacunar infarction identified in our study remained significant after adjusting for these risk factors, suggesting that the association is independent of these factors. Alternatively, the association between plasma TMAO levels and CVSD determined by the WMHV and lacunar infarction demonstrated in our study could be through a mechanism of TMAO via endothelial dysfunction. Endothelial dysfunction is a common pathophysiology that occurs in both large vessel atherosclerosis and small vessel disease. In addition, atherosclerosis and cerebral small vessel pathology often coexist. As mentioned previously, TMAO may be a marker of atherosclerosis as well as CVSD. However, the estimated value of TMAO in CVSD requires further study.

The strengths of this study include the detailed phenotyping of both clinical characteristics and neuroimaging features. Furthermore, the method of metabolite measurement by our group, which uses a targeted metabolomic approach, allows the detection of metabolites with high sensitivity and specificity.^22,23^

### Study Limitations

This study has limitations. This study is a retrospective analysis. Therefore, the associations identified in this study do not necessarily suggest a causal relationship. Future experimental studies are required to fully assess the biological mechanism underlying the relationship between TMAO and CVSD. We also acknowledge the relatively lower prevalence of lacunar infarction in this cohort and the method of WMH detection using 1.5T MRI, which could underestimate the volume of WMH. However, the association between TMAO and WMHV and acute lacunar infarction identified in our study supports a connection between TMAO and CVSD.

## Conclusions

The findings of this cross-sectional study suggested that plasma TMAO levels are associated with both clinical and imaging markers of cerebrovascular small vessel disease in patients with acute ischemic stroke. The association was independent of traditional vascular risk factors.

## Notes

Supplement 1. **eTable.** Baseline Characteristics Comparing Between Entire Study Population With MRI Subgroup

Supplement 2.
Data Sharing Statement
