# Supplementary material for: Trimethylamine N-Oxide and White Matter Hyperintensity Volume Among Patients With Acute Ischemic Stroke
Source: JAMA Netw Open. 2023 Aug 23;6(8):e2330446. doi: 10.1001/jamanetworkopen.2023.30446 (PMC10448304; doi:10.1001/jamanetworkopen.2023.30446)
Supplement: Supplement 1. — eTable. Baseline Characteristics Comparing Between Entire Study Population With MRI Subgroup [file jamanetwopen-e2330446-s001.pdf]

Supplemental Online Content

Kijpaisalratana N, Ament Z, Bevers MB. Trimethylamine-N-oxide and white matter hyperintensity volume and stroke subtype. *JAMA Netw Open*. 2023;6(8):e2330446. doi:10.1001/jamanetworkopen.2023.30446

eTable. Baseline Characteristics Comparing Between Entire Study Population With MRI Subgroup

This supplemental material has been provided by the authors to give readers additional information about their work.

© 2023 Kijpaisalratana N et al. *JAMA Network Open.*

eTable. Baseline Characteristics Comparing Between Entire Study Population With MRI Subgroup

Characteristics	All (351)	MRI subgroup (291)	*p* value
**Age, years, mean (SD)**	69 (15)	67 (15)	0.21
**Male, N(%)**	209 (59.54)	178 (61.17)	0.68
**Stroke risk factors, N(%)**			
**- Hypertension**	255 (72.65)	212 (72.85)	0.95
**- Diabetes mellitus**	75 (21.37)	58 (19.93)	0.66
**- Coronary artery disease**	99 (28.21)	76 (26.12)	0.55
**- Current smoker**	64 (18.29)	58 (19.93)	0.60
**- Hyperlipidemia**	169 (48.15)	140 (48.11)	0.99
**- Prior stroke**	70 (19.94)	57 (19.59)	0.91
**Stroke subtypes, N(%)**			0.91
**- Large vessel atherosclerosis**	68 (19.37)	63 (21.65)	
**- Small vessel disease**	27 (7.69)	26 (8.93)	
**- Cardioembolic**	175 (49.86)	137 (47.08)	
**- Other**	18 (5.13)	15 (5.15)	
**- Undetermined**	63 (17.95)	50 (17.18)	
**Admission NIHSS, median [IQR]**	6 [3,13]	5 [2,12]	0.14
**90-day mRS, median [IQR]**	2 [1,4]	1 [1,3]	0.21
**WMH, median [IQR]**	-	3.2 [1.31,8.4]	-

[a]Number of missing observations for each variable (n): age (6), smoke (1), admission NIHSS (1), mRS (40)

© 2023 Kijpaisalratana N et al. *JAMA Network Open.*
