# Supplementary material for: Trimethylamine N-Oxide and White Matter Hyperintensity Volume Among Patients With Acute Ischemic Stroke
Source: JAMA Netw Open. 2023 Aug 23;6(8):e2330446. doi: 10.1001/jamanetworkopen.2023.30446 (PMC10448304; doi:10.1001/jamanetworkopen.2023.30446)
Supplement: Supplement 2. — Data Sharing Statement [file jamanetwopen-e2330446-s002.pdf]

Data Sharing Statement

Data
Data available: Yes
Data types: Deidentified participant data
How to access data: The data for this study is available to qualified investigators upon reasonable request to the corresponding author (wtkimberly@mgh.harvard.edu).
When available: With publication

Supporting Documents
Document types: None

Additional Information
Who can access the data: anyone requesting the data
Types of analyses: for any purpose
Mechanisms of data availability: with a signed data access agreement
